# Terzyme: a tool for identification and analysis of the plant terpenome

**DOI:** 10.1186/s13007-017-0269-0

**Published:** 2018-01-10

**Authors:** Piyush Priya, Archana Yadav, Jyoti Chand, Gitanjali Yadav

**Affiliations:** 1 Computational Biology Laboratory, National Institute of Plant Genome Research, Aruna Asaf Ali Marg, New Delhi, 110067 India; 2 Department of Plant Sciences, University of Cambridge, Downing Site, Cambridge, CB2 3EA UK

**Keywords:** Terpenome, Terpene synthase (TPS), Prenyl transferase (PT), Hidden Markov Models (HMM), GO clustering, Pathway mapping, Phytochemicals

## Abstract

**Background:**

Terpenoid hydrocarbons represent the largest and most ancient group of phytochemicals, such that the entire chemical library of a plant is often referred to as its ‘terpenome’. Besides having numerous pharmacological properties, terpenes contribute to the scent of the rose, the flavors of cinnamon and the yellow of sunflowers. Rapidly increasing -omics datasets provide an unprecedented opportunity for terpenome detection, paving the way for automated web resources dedicated to phytochemical predictions in genomic data.

**Results:**

We have developed Terzyme, a predictive algorithm for identification, classification and assignment of broad substrate unit to terpene synthase (TPS) and prenyl transferase (PT) enzymes, known to generate the enormous structural and functional diversity of terpenoid compounds across the plant kingdom. Terzyme uses sequence information, plant taxonomy and machine learning methods for predicting TPSs and PTs in genome and proteome datasets. We demonstrate a significant enrichment of the currently identified terpenome by running Terzyme on more than 40 plants.

**Conclusions:**

Terzyme is the result of a rigorous analysis of evolutionary relationships between hundreds of characterized sequences of TPSs and PTs with known specificities, followed by analysis of genome-wide gene distribution patterns, ontology based clustering and optimization of various parameters for building accurate profile Hidden Markov Models. The predictive webserver and database is freely available at http://nipgr.res.in/terzyme.html and would serve as a useful tool for deciphering the species-specific phytochemical potential of plant genomes.

**Electronic supplementary material:**

The online version of this article (10.1186/s13007-017-0269-0) contains supplementary material, which is available to authorized users.

## Background

Modern plants have adapted to the sessile nature of life on land by evolving mechanisms for chemical communication and defence, mediated via low molecular weight compounds, often with complex structures, which have the ability to function in diverse physiological, developmental and evolutionary processes [1]. These phytochemicals, grouped together as plant secondary metabolites, have diversified in both structure and function via gene duplications followed by sub-functionalisation and positive selection for metabolite expansion, such that each species has its unique arsenal of secondary metabolites, many of which are of great significance to humans [2, 3].

Isoprenoids or ‘terpenoids’ represent the largest, most ancient group of phytochemicals, and the entire chemical library of a plant is often referred to as the ‘terpenome’ [4]. Well-known terpenoids include citral, menthol, camphor, cannabinoids and the curcuminoids found in turmeric and mustard seeds. Biosynthesis of terpenes requires the condensation of universal precursor C_5_ isoprene units to form C_15_ or C_20_ prenyl diphosphates (PDPs), catalyzed by short chain prenyl transferase (PT) enzymes, followed by multi-step cyclization reactions catalysed by a huge family of unique enzymes called the terpene synthases (TPSs) [5, 6]. TPSs catalyze one of the most complex reactions known to chemistry and biology, wherein, hundreds of regio- and stereo-specific products can be made from a single substrate by binding and steering polyisoprene substrates through a precise, multistep cyclization cascade that is initiated by the propagation of a highly reactive carbocation [7, 8]. Both PTs and TPSs have a distinct ‘terpene fold’ composed largely of inert amino acids (aa) lining a central active site [9]. They can exhibit very high specificity in product formation with remarkable stereochemical precision [10], as well as huge chemical promiscuity [11]. Molecular investigations of TPSs are an active area of research from the perspective of metabolic engineering. TPSs have been identified and characterized in model plant species of commercial and agronomic value such as *Arabidopsis thaliana* [12], *Citrus* [13], *Vitis vinifera* [14] and *Solanum lycopersicum* [15], as well as in various gymnosperms [16–18].

The TPS gene family in plants reveals functional diversification with members showing clear divergence in different lineages despite similar sequences and structures [19]. This makes it quite challenging to assign substrate specificity to a newly annotated TPS sequence [20]. TPSs have been classified according to two major classification schemes; one based on their functional roles and product formation, whereas the other is based entirely on sequence homology. As per the former scheme, TPSs are divided into three subclasses, namely, monoterpene synthases (Mono-TPSs), sesquiterpene synthases (Sesqui-TPSs), and diterpene synthases (Di-TPSs), depending upon the number of isoprene units condensed by the enzyme, which may be two, three or four, respectively. In terms of protein length, Di-TPSs are the longest (> 850aa) as compared to monoterpene synthases (ranging from 600 to 650aa), and sesquiterpene synthases (between 550 and 580aa long), and this difference arises from an interspersed sequence element in Di-TPSs, conserved both in location and amino acid composition [21]. According to the second TPS classification scheme, seven families are recognized currently, from TPSa to TPSg, with the original clades of TPSe and TPSf merged into a single TPS-e/f subfamily [19, 20, 22]. Of these, the TPSc clade is proposed to be the most ancient, and contains mono- and bifunctional copalyl diphosphate synthase (CPS) proteins from gymnosperms as well as angiosperms. TPSd clade is specific to gymnosperms while the TPSa, b and g subfamilies are angiosperm-specific. TPS-e/f combines the sister subclade-e (representing kaurene synthase B) and its derivative subclade-f that contains linalool synthases, hypothesized to be dicot-specific [20, 22]. From a physiological viewpoint, TPSe and TPSc subfamily members including the (−)-CPS synthases are distantly related to primary metabolism while TPSa, b, and d gene subfamilies are involved in secondary metabolism and show greater diversification.

In this work, we present a comprehensive attempt to identify and classify the PT and TPS gene families in 42 plant species for which nuclear genome sequence data is available in the public domain, leading to the development of Terzyme, an interactive online webserver and database for predictive identification and analysis of the plant terpenome. We also present a detailed computational analysis that was undertaken to assess TPS gene distribution patterns, domain organization and potential functional roles, in order to understand the evolution of novel biochemical functions in different lineages, and to unravel the complexity of the plant terpenome. Assessment of genome wide distribution patterns as well as clustering among the genes of the identified TPSs is important in view of the fact that plants are well known for the occurrence of both genic and chromosomal duplications that have resulted in the widespread existence of gene families in this kingdom, apart from being associated with subsequent evolutionary divergence via sub-functionalization or neo-functionalization [23]. We hope that Terzyme will provide insights into the concept of lineage-specific expansion in the PT and TPS families in various plant species together with their functional roles and to understand the evolution of terpene biosynthetic machinery in plants.

## Results

### Annotated terpenome data

The curated terpenome data was compiled as described in methods, involving retrieval of sequences from the NCBI Protein Database via keyword specific search for prenyl transferases (PTs) as well as all TPS functional classes (mono-, di- and sesqui-TPSs) as well as the sequence homology based gene-family classes, namely TPSa to TPSg [19]. The function-based (FB dataset) consisted of 401 representatives sequences, including 154 monoterpene synthases, 71 diterpene synthases and 176 sesquiterpene synthases, as shown in Table 1. These sequences represent diverse taxonomic classes of green plants, including land plants, which further include seed plants, with the exception of chlorophytes. For prenyl transferases, a total of 301 PT sequences were compiled as shown in the last column of Table 1, and this data was called the PT dataset. Mosses have not been reported to have any prenyl transferases at all. Additional file 1: Table 1 provides a detailed list of accession IDs for each sequence used in the FB and PT datasets. The gene-family based GB dataset was also compiled as described in methods and Table 2 shows the 326 sequences retrieved for this dataset. A detailed list of accession IDs for each sequence, along with species and sub-class information has been provided in Additional file 2: Table 2. As can be seen in Table 2, this dataset contains 113 TPSa, 49 TPSb, 35 TPSc, 48 TPSd, 50 TPSe_f and 31 TPSg sequences, mainly present in seed plants. No TPS has been annotated to date in chlorophytes. Lower plants like ferns and mosses also have very few PTs or TPSs. Similarly, genomes of ancient land plants like the magnoliales also appear to lack PTs or TPSs as per their current annotations. As expected, only gymnosperm sequences are present in the TPSd subfamily, known to be specific to this clade. In all, the FB, GB and the PT datasets consist of 116, 74 and 112 species respectively, the majority being monocots or dicots.

**Table 1:** Data representing the known function-based TPS subfamily (FB) and prenyl transferase family dataset

Plant domain (#plants)	MonoTPSs	DiTPSs	SesquiTPSs	Total (FB)	PT (#plants)
Chlorophytes	–	–	–	–	15 (7)
Bryophytes	–	–	–	–	–
Pteridophytes (1)	–	4	–	04	2 (1)
Gymnosperms (18)	49	26	10	85	14 (6)
Ancient Angiosperms (3)	05	–	2	07	2 (2)
Monocots (14)	11	16	34	61	29 (10)
Eudicots (80)	89	25	130	244	239 (86)
Total (116)	154	71	176	401	301 (112)

#Number of species per category provided in brackets. For details, see supplementary data

**Table 2:** TPS data representing the known gene family based (GB) dataset

	TPSa	TPSb	TPSc	TPSd	TPSe_f	TPSg	Total
Chlorophytes (0)	–	–	–	–	–	–	–
Bryophytes (1)	–	–	01	–	–	–	01
Pteridophytes (1)	–	–	03	–	–	–	03
Gymnosperms (8)	–	–	02	48	02	–	52
Monocots (13)	34	4	08	–	17	03	66
Eudicots (51)	79	45	21	–	31	28	204
Total (74)	113	49	35	48	50	31	326

For details, see supplementary data

### Profile hidden Markov models and predictive accuracy

Profile HMMs were built for the prenyl transferase family and all 12 classes of TPS subfamilies, as described in methods. For TPSs, analysis was divided into two parts, function based analysis and gene family based analysis. In function-based analysis the input sequence is classified into a monoterpene, diterpene or sesquiterpene synthase using the six profile HMMs specific to function based (FB) dataset. In gene family based (GB) analysis, the test sequence is assigned to one of the six gene families described earlier from TPSa to g, with HMMs being generated from the GB dataset. In order to test the predictive accuracy of the program, benchmarking was done as described in methods, and this revealed a sensitivity of 100% in all cases, albeit with a relatively low accuracy range of 51–61%. This indicates that although each individual sub-family search profile is able to successfully identify true positives, the twelve HMMs also have a tendency towards false positives, i.e. acquisition of TPS sequences from other sub-families. The high rate of false positives severely affects sub-family annotation towards prediction of substrate specificity and may be ascribed to the strong homology between various TPS sub-classes, both at sequence and structural levels. Table 3 provides a sense of this overlap through an inter-family sequence and structural fold comparison. The various TPS sub-family representatives used for structural superimposition included the 1,8-cineole synthase from *Salvia fruticosa* for Monoterpene synthases (PDBID 25JC), the Taxadiene synthase from Pacific yew for Di-TPSs (PDB ID 3P5P) and the 5-epi aristolochene synthase from *Nicotiana tabacum* (PDBID 3M00) representing sesqui-TPSs. As a result, correct annotation and classification of newly identified TPSs becomes a significant challenge in view of conservation between the different TPS superfamilies. In order to overcome this challenge posed by high false positives without losing out the perfect sensitivity achieved by each sub-family profile, we developed a pipeline wherein all twelve profile HMMs would be allowed to scan a new sequence in parallel. We then based the final sub-family assignment on the premise that high sensitivity of the true sub-family profile would overshadow the false positive scores of the remaining eleven non-self HMMs. In this manner, even though a given test sequence may be identified by multiple sub-family HMMs, the highest scoring hit would still remain the true sub-family profile. In order to test the veracity of our selection premise, all 12 HMMs were combined into a pipeline for scanning the test set dataset, followed by sorting based on highest score obtained by each sub-family profile. As expected, benchmarking of the program in this manner significantly enhanced the accuracy of the search algorithm as can be seen in Table 4. This table shows the results of predictive performance of HMMs calculated using the statistical concepts of sensitivity and accuracy as discussed in methodology section, before and after the parallel-scan strategy. As can be seen from Table 4, the final accuracy of the search algorithm increases to 100% for a majority of sub-families, with the exception of dicot-specific profiles for Mono- and Sesqui-TPSs, both of which show accuracy above 85%.

**Table 3:**
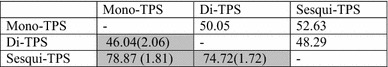
Structural and sequence similarity between TPS sub-families

Unshaded cells represent average %Similarity between members of one subfamily with another while shaded cells represent structural overlap values with RMSD values in brackets. The PDB IDs used for inter-subfamily structural comparison are 25JC (Mono-TPS), 3P5P (Di-TPS) and 3M00 (Sesqui-TPS), representing the 1,8-cineole synthase from *Salvia fruticosa*, Taxadiene synthase from *Pacific yew*, and the 5-epi aristolochene synthase from *Nicotiana tabacum* respectively

**Table 4:** Predictive performance of the 12 profile HMMs (in %)

Category of terpenome prediction models	Sensitivity	Accuracy	Accuracy after combining HMMs
Monoterpene synthases in dicots (MonoD)	100	61	88
Monoterpene synthases in monocots (MonoM)	100	51	100
Diterpene synthases in dicots (DiD)	100	53	100
Diterpene synthases in monocots (DiM)	100	51	100
Sesquiterpene synthases in dicots (SesD)	100	61	86
Sesquiterpene synthases in monocots (SesM)	100	52	100
TPSa	100	60	100
TPSb	100	55	100
TPSc	100	52	100
TPSd	100	54	100
TPe_f	100	54	100
TPSg	100	52	100

Encouraged by the superior predictive power of the search algorithm, we proceeded to assess its performance in context with other online web resources. To our knowledge, there is no search tool specific to any category of phytochemicals, but we expected the global annotation databases, (such as Pfam, PANTHER and Interpro) to be able to identify terpene synthase family, and therefore adequate for a comparative performance test. However, we found that none of the currently existing programs could classify TPSs either based on their function i.e. into monoterpene, diterpene or sesquiterpene synthases, nor on the basis of gene family (TPSa-TPSe/f). The Terzyme hidden markov models, in contrast, achieve taxon-based distinction between identified TPSs. One reason for the failure of general annotation databases may be that available programs like Pfam identify TPSs by detecting either the N-terminal domain naming them as Terpene_synth (PF01397) or the metal binding domain designating it as Terpene_synth_C (PF03936) or both. Similarly PANTHER detects N-terminal domain designating it as PTHR31376, other Terpenoid synthase as PTHR31225, PTHR11439 or PTHR31739. Interpro also detects Terpenoid synthase, N terminal domain as IPR001906 and C-terminal metal binding domain as Terpene_synth_C (IPR005630). Hence, from a predictive viewpoint, it can be inferred that our search algorithm performs better than existing programs for TPS gene family identification in the plant kingdom.

### Novel terpenome identification

**Fig. 1:**
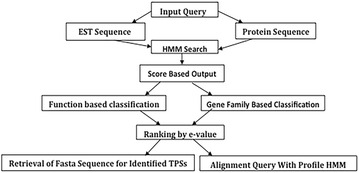
The workflow of terpenome search algorithm

**Fig. 2:**
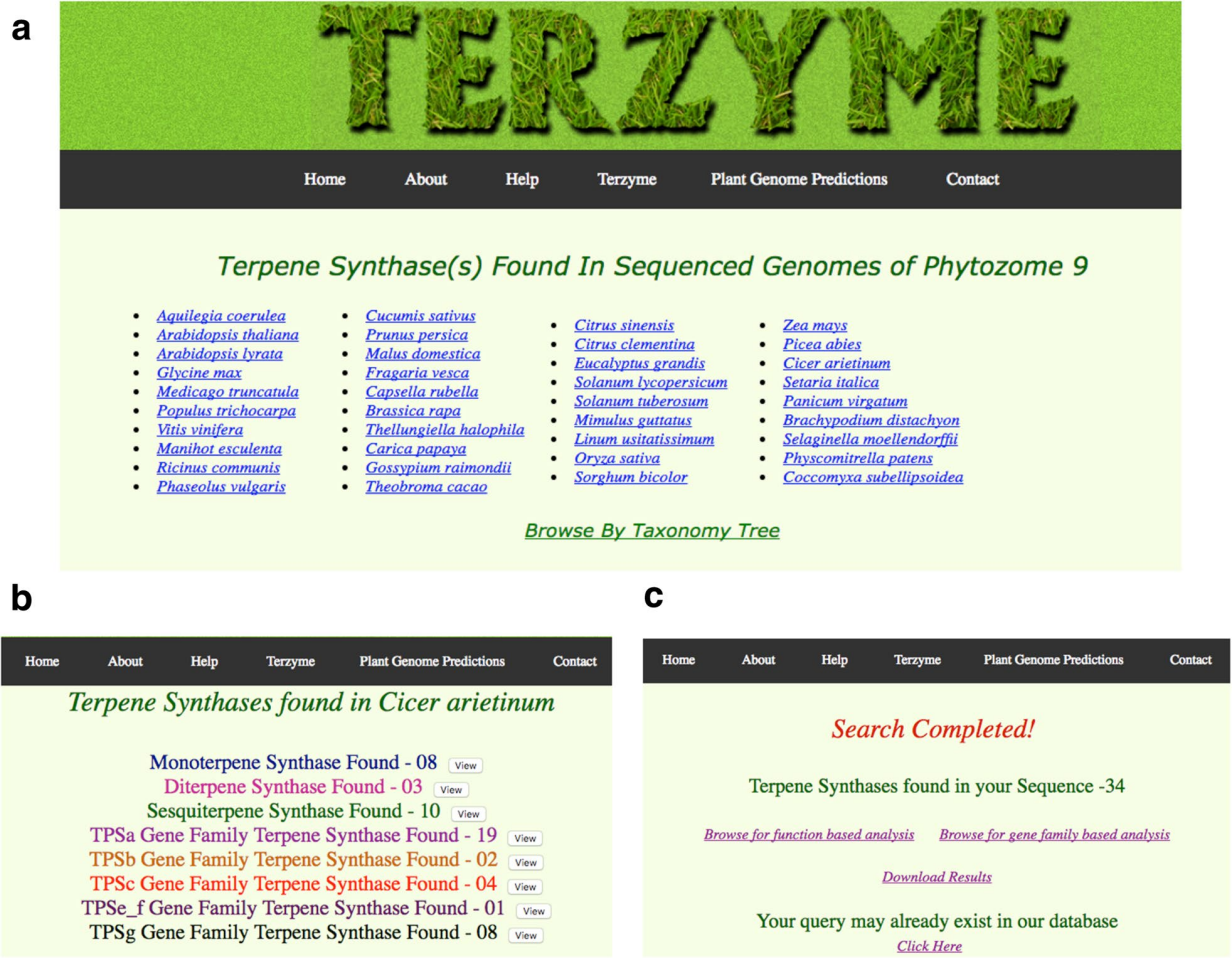
Screenshots of the Terzyme Web resource developed for terpenome identification and annotation. **a** shows the browse-able list of 42 plant species on which the program was scanned. Clicking any of these 42 species links will return the outcome of Terzyme for that plant through a screen similar to **b**, which depicts the putative TPSs identified in the cold season food legume chickpea. Users can also submit their own sequence/s to the Terzyme prediction server and **c** shows a typical outcome for a query

Encouraged by this superior accuracy of prediction, we automated the TPS and PT search pipeline to design and develop the Terzyme interactive online server, available freely without any login requirement at www.nipgr.res.in/terzyme.html. Figure 1 shows the query submission protocol of Terzyme. It has been configured to accept multiple fasta sequences to search for TPSs, and does not require download on local machines for processing. The Terzyme HMM pipeline was used to search for novel TPS and PT gene family members in the entire PSG dataset (containing 1,573,395 protein sequences from 42 plant genomes, as described in Methods), and the resulting identifications have been incorporated into the online web resource for browsing, download and further exploration. Figure 2 shows a few screenshots of the Terzyme prediction server including the browse-able terpenome database. In all, Terzyme identified a total of 3312 unique TPS sequences and 873 unique prenyl transferases. Some of the TPS sequences, as expected, were predicted by both function-based and gene-family based HMMs. These 3312 TPSs and 873 PTs are available for browsing, both by species name and taxonomic class, through the ‘Plant Genome Predictions’ menu of Terzyme website, as shown in panels B of Fig. 2. For each TPS, users can view or download the corresponding sequence in FASTA format, its alignment with the respective sub-family profile HMM, as well as the predicted secondary structure.

A total of 2040 TPSs were identified by the functional class based profile HMMs and these included 613 monoterpene synthases, 468 diterpene synthases and 959 sesquiterpene synthases. Similarly, 2987 TPSs were identified by gene family based profile HMMs which included 1797 TPSa, 432 TPSb, 218 TPSc, 81 TPSd, 270 TPSe_f and 189 TPSg gene family sequences. A complete list of these identifications along with sub-family assignment for each species, including PTs is provided in Tables 5, 6 and 7 respectively. Interestingly, our data shows identification of putative TPSs in three chlorophyte genomes even though none of the profile HMMs were trained on these species. A manual inspection of each sequence shows them to have sufficient length and presence of the requisite TPS motifs. From a functional point of view, all chlorophyte TPSs appear to be Diterpene synthases, the ancient TPS containing family, known previously to consist of both gymnosperm and angiosperm members. A detailed analysis of some of these DiTPSs indicated them to be closely related to Cycloartenol synthase and some were found to contain Squalene cyclase (SQCY) found in class II TPSs. Among bryophytes, *Physcomitrella patens* shows only one known bifunctional TPSs with both CPS/KS activity has been reported till date [24]. The present analysis reveals the additional presence of at least nine bifunctional TPSs, and we have identified 15 prenyl transferases in the moss genome, suggesting a reasonably large terpenome family with more than 30 members, majority of these being previously unreported diterpene synthases. The gene family profiles assign most of these sequences to the TPSa and TPSc subfamilies. As anticipated, 64 of the 72 putative TPSs identified in the gymnosperm genome (*Picea abies*) were assigned to TPSd subfamily, supporting the existing view that gymnosperm TPSs belong to a distinct clade [19]. Tables 5 and 6 also depict five gymnosperm TPS sequences representing the most ancient TPSc gene family, with mono/bifunctional CPSs, although we did not observe any bias of representation in case of functional class assignment; all three classes namely monoTPS, diTPS and sesqui-TPS are roughly equally present in this gymnosperm. It may be noted that Terzyme enables a distinction between Class II Copalyl diphosphate synthases (CPS) and the Class-I Kaurene synthases (KSL) also. Under the Plant Genome Predictions Tab, apart from the Diterpene TPS classification, for each genome, Terzyme shows the exact number of matches found for TPSc and TPSe_f classes, both of which represent largely, the CPS and KSL respectively. We believe this is a very useful feature that enables users to breakdown Di-TPS data for detecting better-resolved functional annotations. In general monoterpene and sesquiterpene synthases outnumber the diterpene synthases in all seed plant domains. This may be due to the general mono functional activity of the former compared to bifunctional activity of latter enzymes. Terpenes are known to play significant roles defence responses against herbivores by emissions of several volatile blends, and volatile emissions are mainly composed of monoterpenes and sesquiterpene lactones owing to their low molecular weights (C_10_ and C_15_ respectively). It may also be noted that the identified TPS gene family size increases from lower plants (chlorophytes, bryophytes and pteridophytes) to land plants, suggesting expansion of the family during course of evolution. An average of 30–50 TPS sequences were identified across higher plants, with the maximum number of sequences detected in *Panicum virgatum* (switchgrass), along with commercial fruit bearing dicot species like apple, grape and papaya. The Eucalyptus genome also contains over one hundred TPS sequences and it would be interesting to study these TPSs further and characterize their roles in the respective genomes. Present knowledge of completely characterized TPSs is limited to only few plant species and their classification based on functional roles or gene family is still an emerging field. Our data on the other hand opens up a huge repertoire of putative TPSs candidates throughout the plant kingdom, together with their functional, and gene family based classification. For example, the tomato terpenome was recently characterized with about 40 TPS [15], whereas our analysis reveals at least 60–100 TPSs in the *S. lycopersicum* genome along with more than 20 PTs. In addition, Diterpene TPS classification has been performed based on class I (Aspartate rich motif (DDXXD/E) or Non-aspartate rich consensus motif of (**N**,**D**)D(L,I,V)X(**S**,**T**)XXX**E** also called as ‘NSE/DTE’ motif and class II (DXDD) signature motifs present in the respective sequences. In house perl script were used to scan all the DiTPS annotations to assess the presence of these signature motifs for Class I and Class II TPSs. Accordingly, TERZYME classifies DiTPSs into the following four classes: (1) Class I DiTPS—If either Aspartate rich motif (DDXXD/E) or Non-aspartate rich consensus motif of (**N**,**D**)D(L,I,V)X(**S**,**T**)XXX**E**, (2) Class II Diterpene synthases—If DXDD motif was present, (3) bifunctional—If both Class I and Class II motifs are present and (4) Noncanonical : If none are present. Details can be seen in Table 8. The presence of these sub-classes in available plants can also be assessed on the Terzyme database under the Plant Genomes Predictions tab. These examples reflect how a rigorous scientific pursuit can lead to new annotations and gene discovery for previously unknown, and even well-known families of conserved sequences.

**Table 5:** Terpenome identified by Function

Plant	Mono TPSs	Di TPSs	SesTPSs	Total TPSs
Chlorophytes				
*Chlamydomonas reinhardtii*	–	02	–	02
*Volvox carteri*	–	01	–	01
*Coccomyxa subellipsoidea*	–	01	–	01
*Micromonas pusilla*	–	–	–	–
*Ostreococcus lucimarinus*	–	–	–	–
Bryophytes				
*Physcomitrella patens*	–	15	–	15
Pteridophytes				
*Selaginella moellendorffii*	04	40	06	50
Gymnosperms				
*Picea abies*	34	17	18	69
Monocots				
Poaceae				
*Oryza sativa*	01	27	39	67
*Sorghum bicolor*	07	05	29	41
*Zea mays*	10	17	29	56
*Setaria italica*	05	13	31	49
*Panicum virgatum*	18	41	56	115
*Brachypodium distachyon*	03	04	13	20
Dicots				
Brassicaceae				
*Arabidopsis lyrata*	12	04	17	33
*Arabidopsis thaliana*	11	04	25	40
*Brassica rapa*	09	06	26	41
*Capsella rubella*	07	04	24	35
*Thellungiella halophila*	03	03	14	20
Malvaceae				
*Gossypium raimondii*	31	11	48	90
*Theobroma cacao*	17	09	28	54
Rosaceae				
*Fragaria vesca*	13	12	34	59
*Malus domestica*	28	24	44	96
*Prunus persica*	11	08	07	26
Rutaceae				
*Citus sinensis*	33	05	44	82
*Citrus clementina*	08	04	01	13
Solanaceae				
*Solanum tuberosum*	09	21	53	83
*Solanum lycopersicum*	26	06	23	55
Fabaceae				
*Glycine max*	19	11	10	40
*Cicer arietinum*	08	03	10	21
*Medicago truncatula*	14	15	17	46
*Phaseolus vulgaris*	19	04	20	43
Salicaceae				
*Populus trichocarpa*	39	11	32	82
Vitaceae				
*Vitis vinifera*	26	09	69	104
Euphorbiaceae				
*Manihot esculenta*	14	13	27	54
*Ricinus communis*	24	11	21	56
Cucurbitaceae				
*Cucumus sativus*	13	05	17	35
Caricaceae				
*Carica papaya*	10	10	17	37
Myrtaceae				
*Eucalyptus grandis*	46	10	58	114
Phrymaceae				
*Mimulus guttatus*	15	25	25	65
Ranunculaceae				
*Aquilega coerulea*	41	13	11	65
Linaceae				
*Linum usitatissimum*	25	24	16	65
Total	613	468	959	2040

**Table 6:** Terpenome identified by gene family

Plant	TPSa	TPSb	TPSc	TPSd	TPSe_f	TPSg	Total
Chlorophytes							
*Chlamydomonas reinhardtii*	–	–	–	–	–	–	–
*Volvox carteri*	–	–	–	–	–	–	–
*Coccomyxa subellipsoidea*	–	–	–	–	–	–	–
*Micromonas pusilla*	–	–	–	–	–	–	–
*Ostreococcus lucimarinus*	–	–	–	–	–	–	–
Bryophytes							
*Physcomitrella patens*	06	–	09	01	02	01	19
Pteridophytes							
*Selaginella moellendorffii*	–	–	14	04	38	–	56
Gymnosperms							
*Picea abies*	01	01	05	64	01	–	72
Monocots							
Poaceae							
*Oryza sativa*	36	01	05	–	15	02	59
*Sorghum bicolor*	28	04	03	–	03	05	43
*Zea mays*	23	06	06	03	07	04	49
*Setaria italica*	33	04	05	–	09	03	54
*Panicum virgatum*	68	08	23	02	20	05	126
*Brachypodium distachyon*	31	05	–	–	04	02	42
Dicots							
Brassicaceae							
*Arabidopsis lyrata*	19	10	01	–	03	02	35
*Arabidopsis thaliana*	29	06	02	–	02	01	40
*Brassica rapa*	37	07	03	–	05	01	53
*Capsella rubella*	38	07	02	–	03	01	51
*Thellungiella halophila*	39	02	01	–	02	–	44
Malvaceae							
*Gossypium raimondii*	46	25	06	02	03	01	83
*Theobroma cacao*	96	15	01	–	05	05	122
Rosaceae							
*Fragaria vesca*	94	11	09	–	03	04	121
*Malus domestica*	174	18	12	–	14	20	238
*Prunus persica*	86	10	02	–	07	02	107
Rutaceae							
*Citus sinensis*	61	32	04	–	04	04	105
*Citrus clementina*	21	05	05	01	06	05	43
Solanaceae							
*Solanum tuberosum*	72	06	18	02	05	06	109
*Solanum lycopersicum*	67	20	03	–	05	05	100
Fabaceae							
*Glycine max*	27	10	08	–	06	07	58
*Cicer arietinum*	19	02	04	–	01	08	34
*Medicago truncatula*	60	09	17	–	05	09	100
*Phaseolus vulgaris*	48	10	05	01	01	09	74
Salicaceae							
*Populus trichocarpa*	57	35	04	01	05	03	105
Vitaceae							
*Vitis vinifera*	78	16	04	–	06	23	127
Euphorbiaceae							
*Manihot esculenta*	61	10	04	–	13	06	94
*Ricinus communis*	34	25	01	–	10	05	75
Cucurbitaceae							
*Cucumus sativus*	35	09	02	–	02	04	52
Caricaceae							
*Carica papaya*	118	10	03	–	11	01	143
Myrtaceae							
*Eucalyptus grandis*	87	23	02	–	09	17	138
Phrymaceae							
*Mimulus guttatus*	32	13	17	–	11	06	79
Ranunculaceae							
*Aquilega coerulea*	20	37	01	–	07	05	70
Linaceae							
*Linum usitatissimum*	16	20	07	–	17	07	67
Total	1797	432	218	81	270	189	2987

**Table 7:** Terpenome identified through prenyl transferase (PT) annotation

Plant	Total PTSs
Chlorophytes	
*Chlamydomonas reinhardtii*	06
*Volvox carteri*	05
*Coccomyxa subellipsoidea*	05
*Micromonas pusilla*	05
*Ostreococcus lucimarinus*	06
Bryophytes	
*Physcomitrella patens*	15
Pteridophytes	
*Selaginella moellendorffii*	16
Gymnosperms	
*Picea abies*	14
Monocots	
Poaceae	
*Oryza sativa*	22
*Sorghum bicolor*	09
*Zea mays*	37
*Setaria italica*	22
*Panicum virgatum*	40
*Brachypodium distachyon*	20
Dicots	
Brassicaceae	
*Arabidopsis lyrata*	20
*Arabidopsis thaliana*	23
*Brassica rapa*	26
*Capsella rubella*	20
*Thellungiella halophila*	19
Malvaceae	
*Gossypium raimondii*	53
*Theobroma cacao*	28
Rosaceae	
*Fragaria vesca*	11
*Malus domestica*	41
*Prunus persica*	11
Rutaceae	
*Citus sinensis*	38
*Citrus clementina*	28
Solanaceae	
*Solanum tuberosum*	31
*Solanum lycopersicum*	21
Fabaceae	
*Glycine max*	31
*Cicer arietinum*	13
*Medicago truncatula*	10
*Phaseolus vulgaris*	14
Salicaceae	
*Populus trichocarpa*	47
Vitaceae	
*Vitis vinifera*	11
Euphorbiaceae	
*Manihot esculenta*	15
*Ricinus communis*	10
Cucurbitaceae	
*Cucumus sativus*	23
Caricaceae	
*Carica papaya*	11
Myrtaceae	
*Eucalyptus grandis*	22
Phrymaceae	
*Mimulus guttatus*	18
Ranunculaceae	
*Aquilega coerulea*	29
Linaceae	
*Linum usitatissimum*	27
Total	873

**Table 8:** Diterpene classification based on Class I and Class II signature motifs

Plant	Class I	Class II	Bifunctional	Unclassified/partial	Total DiTPSs
Chlorophytes					
*Chlamydomonas reinhardtii*	–	–	–	02	02
*Volvox carteri*	–	–	–	01	01
*Coccomyxa subellipsoidea*	–	–	–	01	01
*Micromonas pusilla*	–	–	–	–	–
*Ostreococcus lucimarinus*	–	–	–	–	–
Bryophytes					
*Physcomitrella patens*	03	04	01	03	15
Pteridophytes					
*Selaginella moellendorffii*	29	03	04	04	40
Gymnosperms					
*Picea abies*	09	02	–	06	17
Monocots					
Poaceae					
*Oryza sativa*	15	04	01	07	27
*Sorghum bicolor*	03	02	–	01	06
*Zea mays*	08	05	03	01	17
*Setaria italica*	07	04	01	01	13
*Panicum virgatum*	18	13	–	10	41
*Brachypodium distachyon*	03	–	–	01	04
Dicots					
Brassicaceae					
*Arabidopsis lyrata*	03	01	–	–	04
*Arabidopsis thaliana*	02	01	–	01	04
*Brassica rapa*	03	01	02	–	06
*Capsella rubella*	03	01	–	–	04
*Thellungiella halophila*	02	01	–	–	03
Malvaceae					
*Gossypium raimondii*	02	02	07	–	11
*Theobroma cacao*	05	–	03	01	09
Rosaceae					
*Fragaria vesca*	02	03	02	05	12
*Malus domestica*	05	03	01	15	24
*Prunus persica*	04	01	–	03	08
Rutaceae					
*Citus sinensis*	02	–	–	01	03
*Citrus clementina*	02	–	01	02	05
Solanaceae					
*Solanum tuberosum*	03	11	–	07	21
*Solanum lycopersicum*	03	–	01	02	06
Fabaceae					
*Glycine max*	05	–	03	03	11
*Cicer arietinum*	01	01	–	01	03
*Medicago truncatula*	03	02	01	09	15
*Phaseolus vulgaris*	01	01	02	–	04
Salicaceae					
*Populus trichocarpa*	06	–	04	01	11
Vitaceae					
*Vitis vinifera*	04	01	02	02	09
Euphorbiaceae					
*Manihot esculenta*	09	–	02	02	13
*Ricinus communis*	08	01	–	02	11
Cucurbitaceae					
*Cucumus sativus*	04	01	–	–	05
Caricaceae					
*Carica papaya*	05	–	01	04	10
Myrtaceae					
*Eucalyptus grandis*	08	01	01	–	10
Phrymaceae					
*Mimulus guttatus*	10	07	06	02	25
Ranunculaceae					
*Aquilega coerulea*	10	–	–	03	13
Linaceae					
*Linum usitatissimum*	10	02	02	10	24
Total					468

Gene ontology analysis for the newly identified TPS genes was carried out in order to further validate our predictions for the plant kingdom, as well as to improve the resolution of functional role prediction, in terms of molecular function or subcellular localization. As described in methods, a total of 2040 TPSs were subjected to ontological analysis and as anticipated, the novel TPSs were found to be enriched in biological process terms like ‘response to stress’, ‘lipid metabolic pathway’ and ‘secondary metabolic process’. More than 900 TPSs were found to be enriched for primary metabolism, although this class of genes is mostly known for secondary/ specialized metabolism. An assessment of these TPSs revealed that they belong to the mono/bifunctional CPS of TPSc and highly divergent TPSe_f gene families. Among molecular function categories, highest enrichment was found for terpene synthase activity, catalytic activity and magnesium ion binding activity, as expected, but in few cases, the GO terms were able to resolve the exact catalytic function for a given TPSs, as in case of hydrolases (37 cases), transferases (65 cases) or protein binding activities. In eight cases, nucleic acid binding was found to be an enriched term and we looked at these cases in more detail in order to understand how TPSs may bind to DNA/RNA to carry out their function. It was interesting to note that nucleic acid binding term was found mainly in case of sesquiterpene synthases in response towards oxidative stress. It may be noted that some TPS genes have previously been known to show single-stranded DNA endo-deoxyribonuclease activity or DNA-directed RNA polymerase activity, and take part in double strand break repair via homologues recombination. Thus, the present eight cases may form part of purine or pyrimidine nucleobase metabolic process. In the sub-cellular compartmentalization category, a majority of mono- and di-TPSs were found to be localized in plastids, as expected, since these are synthesized by the methyl erythritol (MEP) pathway, which is plastidial in nature. In contrast, the sesquiterpene synthases were predominantly found to be located in cytoplasm, the site of occurrence of the mevalonic acid (MVA) pathway that is known to synthesize sesquiterpene and triterpenes. 134 cases did not follow the expected localization trend, wherein a sesqui-TPS was localized to the plastid and, conversely a mono- or di-TPS was predicted to be localized in the cytoplasm, supporting the notion of crosstalk that has been previously hypothesized between MVA and MEP pathways [7]. In summary, the GO analysis further supports Terzyme predictions and the huge repertoire of new TPSs thus identified provides an opportunity for further functional characterization. Further analyses, as presented in the next section were performed to shed light on the roles and identities of these new TPSs.

### Analysis of the plant terpenome

**Fig. 3:**
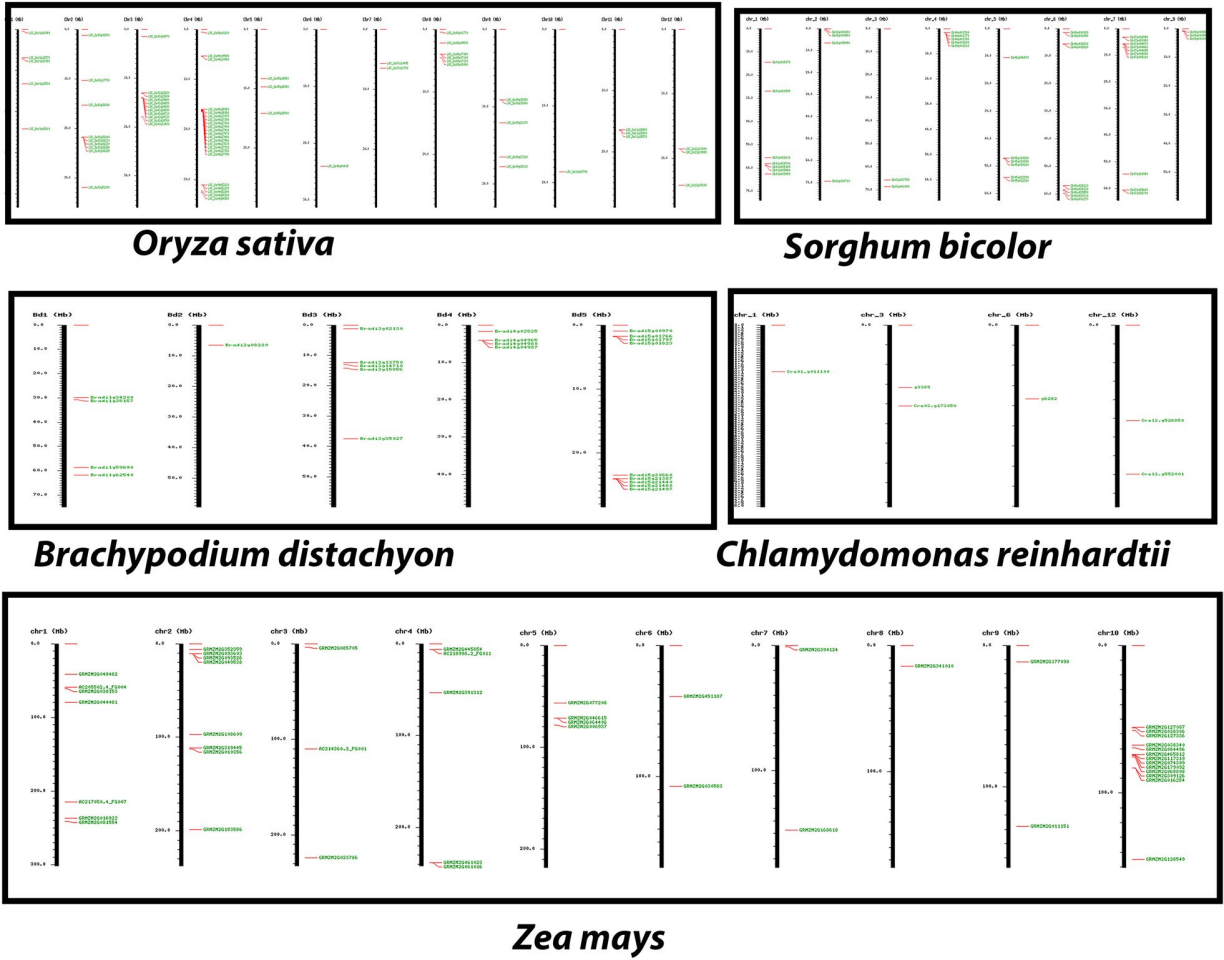
Genome-wide maps of TPSs identified in selected monocots and the chlorophyte *Chlamydomonas reinhardtii*. Clustering is evident in several cases, as discussed in text. All images generated using IGMAP [25] server

**Fig. 4:**
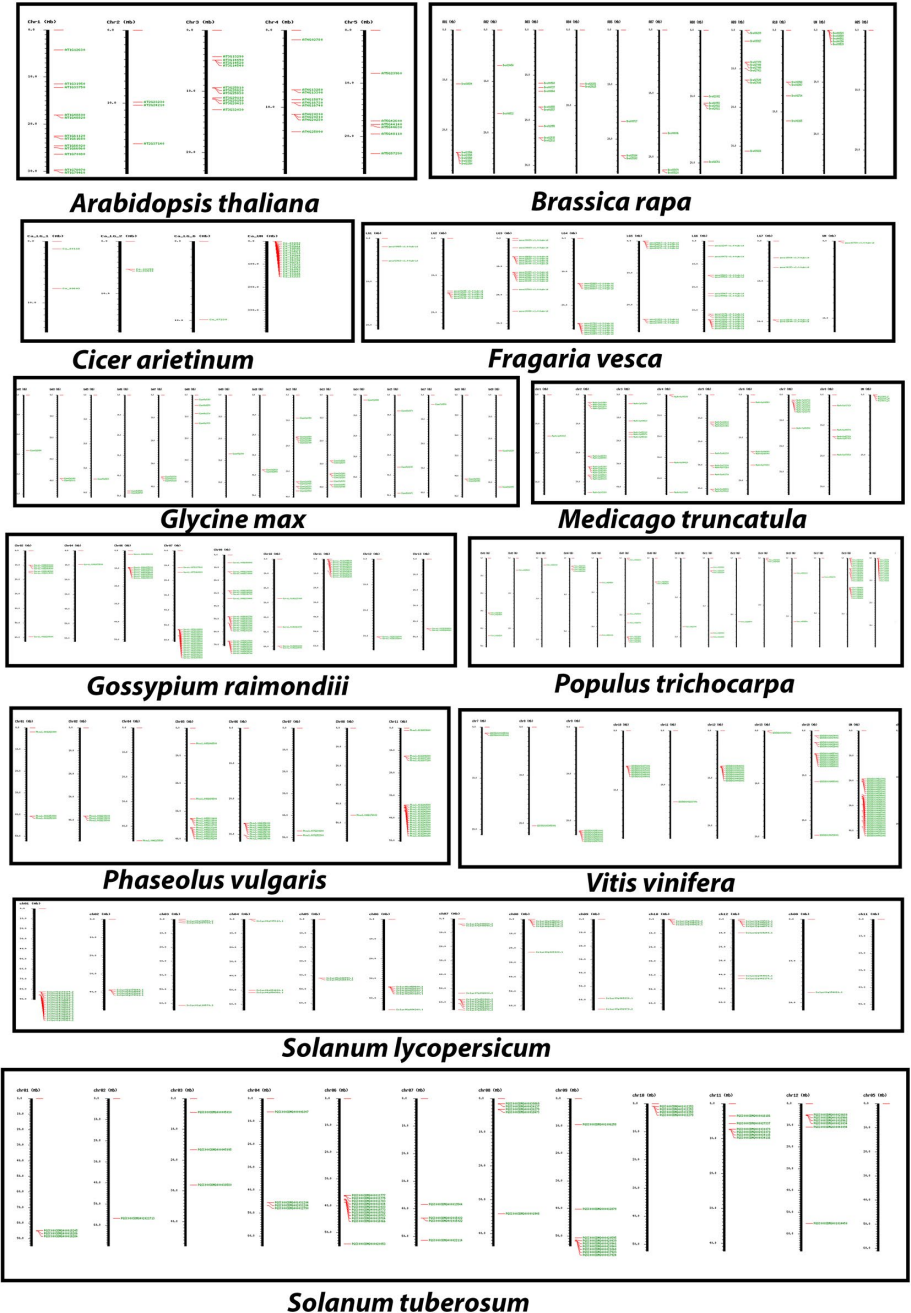
Genome wide maps of TPSs in selected dicot species, discussion in text. Images generated using IGMAP [25] server, developed in our laboratory

The newly identified TPS sequences were subjected to clustering, genome wide mapping and KEGG pathway analysis for understanding their evolution and also for assignment of substrate specificity, as described in Methods. Following GO assignment, the sequences with GO annotations were subjected to EC (Enzyme code) mapping and novel TPSs were mapped onto KEGG pathways, in order to assign putative catalytic roles. In this manner, 539 TPSs were assigned to specific enzymatic categories as shown in Table 9. These included 140 monoterpene synthases, 311 diterpene synthases and 88 sesquiterpene synthases. As can be seen from this table, ent-kaurene synthases were found in the highest number, followed by ent-copalyl diphosphate synthases, both DiTPSs. One each of bornyl-diphosphate synthase, levopimaradiene synthase and germacrene A synthase were predicted, these three representing one each of a MonoTPS, DiTPS and SesquiTPS respectively. Detailed information on accession IDs and substrate preferences for each of these 539 putative TPS sequences is provided in Additional file 3: Table 3. The IGMAP tool [25] was used for clustering the TPSs mapped to 19 plant genomes. Figure 3 depicts the genome-wide terpenome maps for selected monocots and a unicellular green alga, while Fig. 4 depicts the corresponding maps for selected dicotyledonous species. TPSs in both taxa can be observed in clusters often as large as 15 genes, with a tendency to be located towards the centromeres, as in case of rice and Arabidopsis, or towards the edges of the chromosomes, as in sorghum, maize, Brachypodium and most dicots. In *Zea mays*, TPS clusters map both towards center and towards the end of chromosomes. Statistical tests reinforced the trend observed in the map images, viz., a significant number of sequences representing TPS genes are located in clusters within the genomes analyzed, based on an unranked independent samples *T*-test at the 99% confidence level (*P* value = 5.52E−08). Clustering data is presented in Table 10, it shows positive correlation bewteen TPS gene family size and the corresponding number of gene clusters.

**Table 9:** Depiction of functional diversity of TPSs using KEGG module

Terpene synthases	Enzyme codes: activity	TPSs mapped
Monoterpene synthases (TPSa and TPSg)	EC 5.5.1.8 bornyldiphosphate synthase	01
	EC 4.2.3.25 (TPS14) (3S)-linalool synthase	20
	EC 4.2.3.15 myrcene/ocimene synthase	45
	EC 4.2.3.16 (4S)-limonene synthase	27
	EC 4.2.3.20 (R)-limonene synthase	47
Diterpene synthases (TPSc and TPSb)	EC 5.5.1.13 ent-copalyldiphosphate synthase	83
	EC 5.5.1.12 copalyldiphosphate synthase	19
	EC 5.5.1.14 syn-copalyl-diphosphate synthase	26
	EC 4.2.3.19 ent-kaurene synthase	97
	EC 4.2.3.28 ent-cassa-12, 15-diene synthase	06
	EC 4.2.3.29 ent-sandaracopimaradiene synthase	12
	EC 4.2.3.30 ent-pimara-8 (14), 15-diene synthase	21
	EC 4.2.3.18 abieta-7, 13-diene synthase	03
	EC 4.2.3.33 stemar-13-ene synthase	16
	EC 4.2.3.34 stemod-13 (17)-ene synthase	18
	EC 4.2.3.35 syn-pimara-7, 15-diene synthase	09
	EC 4.2.3.32 levopimaradiene synthase	01
Sesquiterpene synthases (TPSb)	EC 4.2.3.23 germacrene-A synthase	01
	EC 4.2.3.22 germacradienol synthase	24
	EC 4.2.3.21 vetispiradiene synthase	18
	EC 4.2.3.13 (+)-delta-cadinene synthase	45
Total TPSs assigned functional roles		539

**Table 10:** TPSs genes clustering across various genomes using IGMAP

Plant	Total TPSs	TPS present within clusters	Clustered genes (%)
Monocots			
Poaceae			
*Oryza sativa*	71	48	67.6056338
*Sorghum bicolor*	46	34	73.91304348
*Zea mays*	47	17	36.17021277
*Brachypodium distachyon*	23	13	56.52173913
Eudicots			
Brassicaceae			
*Arabidopsis thaliana*	42	27	64.28571429
*Brassica rapa*	47	25	53.19148936
Malvaceae			
*Gossypium raimondii*	76	62	81.57894737
Rosaceae			
*Fragaria vesca*	60	46	76.66666667
Solanaceae			
*Solanum lycopersicum*	63	46	73.01587302
*Solanum tuberosum*	89	76	85.39325843
Fabaceae			
*Glycine max*	45	24	53.33333333
*Cicer arietinum (desi)*	25	2	8.00
*Medicago truncatula*	52	31	59.61538462
*Phaseolus vulgaris*	49	41	83.67346939
Salicaceae			
*Populus trichocarpa*	65	35	53.84615385
Vitaceae			
*Vitis vinifera*	118	83	70.33898305
Clustering significance (unranked *T*-test)			*P* < 5.52302E − 08

## Discussion

Plant essential oils are complex mixtures of volatile organic compounds, which play indispensable roles in communication, defense, and adaptive evolution. The complete chemical library produced by a plant is referred to as its terpenome. One way of measuring the terpenome is through knowledge-based prediction of the biosynthetic machinery that generates the enormous diversity of these hydrocarbons, and this method has gained popularity in recent years for various gene families, with the advent of large-scale genome sequencing technologies. In this work we have used this method to identify about 4000 putative PTs and TPSs, representing a huge expansion of the hitherto known plant terpenome. Specifically, 2132 and 2957 function based and gene family based TPSs as shown in Tables 5 and 6, of which, 3312 sequences were unique. These TPSs were assigned to various sub-classes and analysed further for functional role prediction, through large-scale genome wide mapping and clustering, GO enrichment and KEGG mapping, resulting in assignment of substrate or product specificity to more than 500 newly identified TPS sequences.

Gene ontology results validated our predictions to a large extent and KEGG pathway analysis was used to identify potential catalytic roles and substrate preference for over 500 TPSs. The plant kingdom has been known for widespread occurrence of genome wide duplication events, leading to the evolution of biosynthetic modules and clustered organization of genes, that have been observed and reported for several major classes of plant based secondary metabolites. One of the first studies in this area reported the existence of operon-like clusters of terpene-biosynthetic pathway genes with characteristic modularity, physical clustering, and co-regulation, evident in cyanogenic glycosides of *A. thaliana* and avenacin triterpenoids in oats [26]. These reports prompted us to develop IGMAP, a novel computational platform for identification, clustering, and interactive mapping of genes, families, and duplications across genomes for annotated NGS data [25]. In this work, we used IGMAP to perform a large scale spatial cluster analysis of novel TPSs that were identified using TERZYME. Spatial cluster analysis via IGMAP enables the identification of clustered arrays of genes on respective chromosomes in various genomes, and whether or not such clustered spatial patterns of genomic positioning are conserved within and between species or taxa. This effort led to generation of genome wide maps of the identified terpenome, which, in turn revealed a significant tendency to cluster, with individual clusters ranging in size from as few as three to over a dozen terpene synthases, as can be seen in Figs. 3 and 4. Spatial cluster analysis also suggested revealed TPSs to be located near the ends of chromosomes or close to centromeric regions, supporting the concept of selective advantage for clustered genes in plants (Figs. 3, 4). Among eudicots, in family Brassicaceae, TPS clusters of two to three genes were present in *A. thaliana* while in *Brassica rapa*, TPS gene clusters comprise of two to five genes. Family Rosaceae represented by *Fragaria vesca* contains tandem clusters of TPSs both in frequency and size (2–7 genes). In Fabaceae however, fewer TPS clusters were observed, also reflected in the terpenome maps of *Glycine max* and *Cicer arietinum*, as compared to *Medicago truncatula* where more distinct gene clusters were found comprising of 2–4 genes. The largest gene clusters were found in case of *Phaseolus vulgaris* chromosome 11, consisting of about 15 genes. It would be interesting to study this cluster further and characterize it in order to check whether this cluster is involved in synthesis of a specific terpenoid compound. The Malvaceae represented by *Gossypium raimondiii* has large TPS clusters; located mainly in chromosomes 2, 6, 7, 9 and 11. *Populus trichocarpa* of family Salicaceae has large TPS clusters on chromosome 19. Further, Vitaceae represented by grape genome was also found to contain several tandem TPS clusters on chromosomes 9, 10, 12 and 19, with two to eight genes in each cluster. In family Solanaceae, both potato and tomato show large TPS clusters of upto 17 genes. *Solanum tuberosum* also has the highest percentage of TPSs genes (85%) present in clusters. Among monocots, *Oryza sativa* showed large TPS clusters in chromosome 2, 3 and 4, which were similar, both in context of numbers of clusters and its size as compared to eudicots. Overall, IGMAP [25] is able to identify tandem duplications, but we believe that a large number of segmental duplications would also be present in these plants, and methods to directly assess extent of segmental duplication would be very useful for such studies. The identified TPS clusters represent a new avenue of research and would serve as excellent models for studying genome plasticity or novel mechanisms of adaptive evolution.

A recent report describing the detection of a sesterterpene biosynthetic repertoire in Brassicaceae through genome mining, offered to us an opportunity to test the performance of Terzyme, apart from statistical benchmarks shown in earlier sections. The published work included characterization of seven new TPS genes of which five are from Arabidopsis, one from *Capsella rubella* and one from *Brassica oleracea* [27]. Terzyme server was able to detect each of the seven STSs, and classified these as sesquiterpene synthases. Furthermore, six of these STSs (namely AT3G14490, AT3G14520, AT3G14520, AT3G32030, AT3G29410 and Carubv10016237m) are already present in our terpenome database, with the exception of the TPS from *Brassica oleracea* since Terzyme only contains data for complete genomes and the *B. oleracea* genome is not in the database. These results further support the suitability of Terzyme as a tool of choice for biologists working in the area of terpenome detection and analysis.

As an interesting offshoot of this study, we are now integrating Terzyme data with existing species-specific transcriptome datasets and cis-regulome records, in order to construct gene regulatory networks that can shed further light on how the terpenome has expanded and evolved in various taxonomic groups (GY unpublished data). In addition, we recently compared the ‘potential’ terpenome (as predicted by Terzyme) with the ‘actual’ terpenome, integrating volatile compound emission data in conjunction with genomic data to understand how a plant creates the so-called final terpenome, specific to itself, and whether or not plants tap the complete potential for terpene biosynthesis at their disposal according to their genomes [28]. Comparison of actual terpenome with the potential terpenome, as performed in this study, revealed how plants modulate their TPSs expression based on condition or environment-specific needs.

## Conclusions

In this work, we describe Terzyme, a new web resource for identification and classification of terpene synthases, towards prediction of TPS and prenyl transferase gene families in a plant genome, followed by a comprehensive large scale assessment of the identified terpenome, based on data from 42 available plant species with complete nuclear genomes. Terzyme represents a collection of profile Hidden Markov Models (HMMs) based on a rigorous analysis of characterized PTs and terpene synthases in plants. Available freely at www.nipgr.res.in/terzyme.html, it is an online, automated, and predictive search tool, for accurate identification and classification of plant terpene synthases and prenyl treansferases, both on the basis of their function and evolutionary relationships. Terzyme has been designed to accept EST input in addition to protein sequences, and this can assist researchers with preliminary annotation of newly emerging NGS data. The Terzyme website has a tutorial section on submission as well as exploratory analysis. Links to the PDB have been provided for all known 3D structures in the family. Over 3000 novel sequences have been identified in this work and analysed further for functional role prediction. The analyses include TPS identification, assignment to functional or gene family based classes, followed by genome wide mapping and clustering of selected novel TPSs. GO enrichment and KEGG mapping were also carried out, to enable assignment of exact catalytic function to more than 500 TPS sequences identified using Terzyme.

Taken together, the present work enables future investigations into several other aspects of the terpenome like rational design or alteration of substrate preferences towards user-desired scent bouquets through genetic engineering. The idea of a potential terpenome, as described here, will aid in determination of the exact range of product complexity of terpenoid hydrocarbons that a given species may be capable of, thereby paving the way for use of plant-derived terpenoids in the development of new pharmaceuticals, and commercial compounds.

## Methods

### Data collection

For identification of the plant terpenome, full genome sequences and TPS protein sequences were collected from the NCBI Protein Database as it includes translations from annotated coding regions in GenBank, RefSeq and TPA, and also records from SwissProt, PIR, PRF, and PDB. TPS sequences were extracted using keyword searches specific to their functional classes. The first dataset comprised of curated sequences belonging to the three major functional classes of TPSs, namely the Monoterpene synthases, Diterpene synthases, and Sesquiterpene synthases. This dataset was called the curated function-based or FB dataset. For identification of the plant prenyl transferases, annotated PT protein sequences were extracted using keyword searches specific to their functional classes i.e. geranyl diphosphate synthase, geranyl geranyl diphosphate synthase and farnesyl diphosphate synthase. Apart from the functional classification (FB dataset) of TPSs, protein sequences were collected for each of the six homology based TPS classes, and this dataset was called the gene family based (GB) dataset. For data on six known GB classes, literature based annotation was used [15, 20]. FB dataset was further divided into two classes, representing monocots and dicots (since our preliminary analysis showed that the predictive performance of the gene family was better when separated into taxon based classes). These six classes of FB data, along with six classes of GB and one PT class, were used for generating 13 profile hidden markov models (HMMs). Protein sequence prediction data was downloaded for 41 sequenced plant genome projects from Phytozome v9.1. Also, for expansion of our analysis across the major taxonomic lineages of the plant kingdom, the available genome sequence of the gymnosperm *Picea abies* was downloaded from its project website ftp://plantgenie.org/Data/ConGenIE/Picea_abies/ [29]. This combined data representing 42 genomes was called the ‘Plant Sequenced Genome’ (PSG) dataset. In summary, the FB, PT and the GB datasets were used for training and testing the HMM based search algorithm, while the PSG dataset was used for identification of novel TPSs.

### TPS search algorithm

Profile HMMs were built using HMMER (Version-HMMER-3.0) [30]. In all, thirteen profile HMMs were developed, one for prenyl transferases, six for function-based class in monocots and dicots and six based on gene family classes. For all the families, the default weighing method, i.e. the Henikoff position-based sequence-weighting scheme was used. Multiple alignments and phylogenetic reconstruction of the sequences in the FB and GB datasets were carried out using CLUSTALX. Structural superimpositions between 3D representatives of various TPS sub-classes was performed at the C-alpha carbon using CLICK tool [31].

### Benchmarking of the program

For program testing and prediction accuracy, a positive test dataset was separated at the time of data collection, comprising 10% of both FB and GB datasets in order to have representatives from all six function based classes as well as the six gene-family based classes. In order to check the precision accuracy of the program, and more importantly its negative prediction ability, a negative dataset comprising closely related sequences was added to the test set for functional classification as well as gene family based classification. Thus the test data set comprised of both negative as well as positive sequences. The predictive performance of all twelve profile HMMs was tested using the statistical concepts of sensitivity and accuracy. Sensitivity measures the proportion of actual positives, which are correctly identified as such, and was calculated for each family as the ratio of true positives to combined true positives and false negatives. Accuracy estimates the overall proportion of true positives in the population. In addition to these parameters, benchmarking of the search algorithm also involved comparison of Terzyme performance with existing global annotation databases like PFAM, PANTHER and Interpro.

### The Terzyme server

The HMMER based analysis pipeline developed for identification of TPSs as described above, was converted into a web server using HTML and back-end CGI coding. All new TPS and PT identifications were also incorporated into the same web resource, designated as Terzyme, available online freely to the scientific community at http://nipgr.res.in/terzyme.html. The Terzyme webserver is compatible across platforms, and has been tested on several browsers and platforms, including Safari, Firefox, Konqueror and IE on Macintosh, Linux as well as Windows workstations. Perl and shell scripts were used to sort the top hits based on highest score obtained from all 13 profile HMMs. If user input is EST data, an additional e-value filter of 0.01 is applied. The EST data is subjected to a six-frame translation using Transeq tool of EMBOSS. For protein sequence data, default e-value is used with all profile HMMs. Shell scripts have been incorporated to accept simultaneous requests from multiple users making Terzyme a more robust platform. To aid further confirmation of predictive results, a protein secondary structure prediction server has been incorporated into the web resource, based on PSIPRED 3.5 [32]. This server can handle multiple fasta queries for efficient performance. The backend of this server used UniRef90 [33] dataset for psiBLAST [34] within PSIPRED runs. The PSIPRED output gets color-coded according to predicted secondary structure elements through in house PERL scripts.

### Analysis of the terpenome

The novel TPSs thus identified were used for downstream analysis of the plant terpenome. For this, we used IGMAP [25], a program developed earlier by our group for genome-wide mapping and clustering studies, including assessment of spatial patterns of newly identified TPSs on the respective genomes and to find out whether clustering patterns indeed exist in plant terpenomes. TPS sequences identified from the PSG dataset as described above, were analyzed further, to gain a better insight into their product complexity. This was done via GO enrichment analysis, phylogenetics and assessment of intron–exon patterns. For gene ontology studies, Blast2GO [35] tool was used to annotate the novel TPSs belonging to various functional classes. The main annotation pipeline of the tool consists of three sequential steps namely: blast, mapping and annotation. Blast2GO uses the Basic Local Alignment Search Tool (BLAST) to find sequences similar to a query set. Retrieval of GO terms associated to the hits obtained from a BLAST search followed this step. The step assigns evaluated set of GO annotations for the input query sequences. Interpro annotations in Blast2GO were used to retrieve domain/motif information for each sequence. In the final step, predicted TPS enzymes were mapped onto corresponding KEGG pathways [35] and enzyme codes were obtained by mapping from equivalent GO’s. This step led to the exploration of the functional diversity of predicted TPSs in terms of their catalytic activity and substrate preferences.

## Additional files

**Additional file 1: Table 1.** Positive Dataset for training profile HMMs. A detailed list of accession IDs for each sequence used in the FB and PT datasets

**Additional file 2: Table 2.** Positive Dataset for training profile HMMs. A detailed list of accession IDs for each sequence used in the GB dataset

**Additional file 3: Table 3.** Detailed information on accession IDs and substrate preferences for 539 putative TPS sequences
